# Supplementary material for: Novel 3′-Substituted-1′,2′,4′-Oxadiazole Derivatives of 18βH-Glycyrrhetinic Acid and Their O-Acylated Amidoximes: Synthesis and Evaluation of Antitumor and Anti-Inflammatory Potential In Vitro and In Vivo
Source: Int J Mol Sci. 2020 May 15;21(10):3511. doi: 10.3390/ijms21103511 (PMC7279002; doi:10.3390/ijms21103511)
Supplement: Supplementary file 1 [file ijms-21-03511-s001.zip › Supplementary NMR HRMS.docx]

Novel 3ʹ-subsituted-1ʹ,2ʹ,4ʹ-oxadiazole derivatives of 18βH-glycyrrhetinic acid and their O-acylated amidooximes: synthesis and evaluation of anti-tumor and anti-inflammatory potential in vitro and in vivo

Andrey V. Markov ^1,^*, Aleksandra V. Sen’kova ^1^, Irina I. Popadyuk ^2^, Oksana V. Salomatina ^1,2^, Evgeniya B. Logashenko ^1^, Nina I. Komarova ^2^, Anna A. Ilyina ^1^, Nariman F. Salakhutdinov ^2^ and Marina A. Zenkova ^1^

^1^ Institute of Chemical Biology and Fundamental Medicine, Siberian Branch of the Russian Academy of Sciences, Lavrent’ev ave., 8, 630090 Novosibirsk, Russia; [alsenko@mail.ru](mailto:alsenko@mail.ru) (A.V.S.), [evg_log@niboch.nsc.ru](mailto:evg_log@niboch.nsc.ru) (E.B.L.), [humanity2206@mail.ru](mailto:humanity2206@mail.ru) (A.A.I.), [marzen@niboch.nsc.ru](mailto:marzen@niboch.nsc.ru) (M.A.Z.)

^2^ N.N. Vorozhtsov Novosibirsk Institute of Organic Chemistry, Siberian Branch of the Russian Academy of Sciences, Lavrent’ev ave., 9, 630090 Novosibirsk, Russia; [popadyuk@nioch.nsc.ru](mailto:popadyuk@nioch.nsc.ru) (I.I.P), [ana@nioch.nsc.ru](mailto:ana@nioch.nsc.ru) (O.V.S.), [komar@nioch.nsc.ru](mailto:komar@nioch.nsc.ru) (N.I.K.), [anvar@nioch.nsc.ru](mailto:anvar@nioch.nsc.ru) (N.F.S.)

***** Correspondence: [andmrkv@gmail.com](mailto:andmrkv@gmail.com) (A.V.M.); Tel.: +7-383-363-51-61

**Supplementary File 1.** NMR Spectrum of synthesized compounds

Spectrum of Compound **3a**, ^1^H NMR, 400MHz, CDCl_3_

Spectrum of Compound **3a**, ^13^C NMR, JMOD, 100MHz, CDCl_3_

High resolution mass spectrum of compound **3a**, Tsource=90°C, Tprobe=200°C

Calculated m/z=568.3871 (C_34_H_52_O_5_N_2_) ^+^

Found m/z=568.3876

Spectra of compound **3a**, ^1^H NMR, 500MHz, CDCl_3_–CD_3_OD (bottom); ^13^C NMR, JMOD, 125MHz, CDCl_3_–CD_3_OD (top)

Spectra of compound **3a**, ^1^H–^1^H 2D NMR, COSY, 500MHz, CDCl_3_–CD_3_OD

Spectra of compound **3a**, ^1^H–^13^C 2D NMR, HSQC, 500MHz, CDCl_3_–CD_3_OD

Spectra of compound **3a**, ^1^H–^13^C 2D NMR, HMBC, 500MHz, CDCl_3_–CD_3_OD

Spectrum of Compound **4a**, ^1^H NMR, 400MHz, CDCl_3_

Spectrum of Compound **4a**, ^13^C NMR, JMOD, 100MHz, CDCl_3_

High resolution mass spectrum of compound **4a**, Tsource=<50°C, Tprobe=260°C

Calculated m/z=550.3765 (C_34_H_50_O_4_N_2_) +∙

Found m/z= 550.3764

Spectrum of Compound **4a**, ^1^H NMR, 500MHz, CDCl_3_

Spectrum of Compound **4a**, ^13^C NMR, JMOD and BB, 125MHz, CDCl_3_

Spectra of compound **4a**, ^1^H–^1^H 2D NMR, COSY, 500MHz, CDCl_3_–CD_3_OD

Spectra of compound **4a**, ^1^H–^13^C 2D NMR, HSQC, 500MHz, CDCl_3_–CD_3_OD

Spectra of compound **4a**, ^1^H–^13^C 2D NMR, HMBC, 500MHz, CDCl_3_–CD_3_OD

Spectrum of Compound **5a**, ^1^H NMR, 400MHz, CDCl_3_

Spectrum of Compound **5a**, ^13^C NMR, JMOD, 100MHz, CDCl_3_

High resolution mass spectrum of compound **5a**, Tsource=70°C, without heating the probe

Calculated m/z= 508.3660 (C_32_H_48_O_3_N_2_)^+^

Found m/z= 508.3651

Spectrum of Compound **5a**, ^1^H NMR, 500MHz, CDCl_3_

Spectrum of Compound **5a**, ^13^C NMR, JMOD and BB, 125MHz, CDCl_3_

Spectra of compound **5a**, ^1^H–^1^H 2D NMR, COSY, 500MHz, CDCl_3_–CD_3_OD

Spectra of compound **5a**, ^1^H–^13^C 2D NMR, HSQC, 500MHz, CDCl_3_–CD_3_OD

Spectra of compound **5a**, ^1^H–^13^C 2D NMR, HMBC, 500MHz, CDCl_3_–CD_3_OD

Spectrum of Compound **3b**, ^1^H NMR, 400MHz, CDCl_3_

Spectrum of Compound **3b**, ^13^C NMR, JMOD, 100MHz, CDCl_3_

High resolution mass spectrum of compound **3b**, Tsource=90°C, Tprobe=230°C

Calculated m/z=582.4027 (C_35_H_54_O_5_N_2_)^+^

Found m/z=582.4026

Spectrum of Compound **4b**, ^1^H NMR, 400MHz, CDCl_3_

Spectrum of Compound **4b** ^13^C NMR, JMOD, 100MHz, CDCl_3_

High resolution mass spectrum of compound **4b**, Tsource=110°C, Tprobe=200°C

Calculated m/z= 564.3922 (C_35_H_52_O_4_N_2_)^+^

Found m/z= 564.3919

Spectrum of Compound **5b**, ^1^H NMR, 400MHz, CDCl_3_

Spectrum of Compound **5b** ^13^C NMR, JMOD, 125MHz, CDCl_3_

High resolution mass spectrum of compound **5b**, Tsource=85°C, Tprobe=310°C

Calculated m/z=522.3816 (C_33_H_50_O_3_N_2_)^+^

Found m/z=522.3820

Spectrum of Compound **3c**, ^1^H NMR, 300MHz, CDCl_3_

Spectrum of Compound **3c** ^13^C NMR, JMOD, 75MHz, CDCl_3_

High resolution mass spectrum of compound **3c**, Tsource=95°C, Tprobe=280°C

Calculated m/z=596.4184 (C_36_H_56_O_5_N_2_)^+^

Found m/z=596.4178

Spectrum of Compound **4c**, ^1^H NMR, 400MHz, CDCl_3_

Spectrum of Compound **4c** ^13^C NMR, BB, 125MHz, CDCl_3_

High resolution mass spectrum of compound **4c**, Tsource=90°C, Tprobe=250°C

Calculated m/z= 578.4078 (C_36_H_54_O_4_N_2_)^+^

Found m/z= 578.4073

Spectrum of Compound **5c**, ^1^H NMR, 500MHz, CDCl_3_

Spectrum of Compound **5c** ^13^C NMR, JMOD, 125MHz, CDCl_3_

High resolution mass spectrum of compound **5c**, Tsource=90°C, Tprobe=250°C

Calculated m/z=536.3973 (C_34_H_52_O_3_N_2_) ^+^∙

Found m/z= 536.3975

Spectrum of Compound **3d**, ^1^H NMR, 500MHz, CDCl_3_

Spectrum of Compound **3d** ^13^C NMR, JMOD, 125MHz, CDCl_3_

High resolution mass spectrum of compound **3d**, Tsource=85°C, Tprobe=250°C

Calculated m/z= 610.4340 (C_37_H_58_O_5_N_2_)^+^

Found m/z= 610.4346

Spectrum of Compound **4d**, ^1^H NMR, 400MHz, CDCl_3_

Spectrum of Compound **4d** ^13^C NMR, BB, 125MHz, CDCl_3_

High resolution mass spectrum of compound **4d**, Tsource=90°C, Tprobe=230°C

Calculated m/z=592.4235 (C_37_H_56_O_4_N_2_) ^+^∙

Found m/z=592.4243

Spectrum of Compound **5d**, ^1^H NMR, 400MHz, CDCl_3_

Spectrum of Compound **5d** ^13^C NMR, JMOD, 100MHz, CDCl_3_

High resolution mass spectrum of compound **5d**, Tsource=95°C, Tprobe=250°C

Calculated m/z=550.4129 (C_35_H_54_O_3_N_2_)^+^

Found m/z=550.4132

Spectrum of Compound **3e**, ^1^H NMR, 500MHz, CDCl_3_

__

Spectrum of Compound **3e** ^13^C NMR, JMOD, 125MHz, CDCl_3_

High resolution mass spectrum of compound **3e**, Tsource=65°C, Tprobe=250°C

Calculated m/z=630.4027 (C_39_H_54_O_5_N_2_)^+^

Found m/z=630.4021

Spectrum of Compound **4e**, ^1^H NMR, 400MHz, CDCl_3_

__

Spectrum of Compound **4e** ^13^C NMR, JMOD, 100MHz, CDCl_3_

High resolution mass spectrum of compound **4e**, Tsource=60°C, Tprobe=250°C

Calculated m/z=612.3922 (C_39_H_52_O_4_N_2_) ^+^∙

Found m/z=612.3929

Spectrum of Compound **5e**, ^1^H NMR, 400MHz, CDCl_3_

__

Spectrum of Compound **5e** ^13^C NMR, JMOD, 125MHz, CDCl_3_

High resolution mass spectrum of compound **5e**, Tsource=55°C, Tprobe=300°C

Calculated m/z= 570.3816 (C_37_H_50_O_3_N_2_) ^+^∙

Found m/z= 570.3814

Spectrum of Compound **3f**, ^1^H NMR, 400MHz, CDCl_3_

__

Spectrum of Compound **3f** ^13^C NMR, JMOD, 100MHz, CDCl_3_

High resolution mass spectrum of compound **3f**, Tsource=85°C, Tprobe=300°C

Calculated m/z= 631.3980 (C_38_H_53_O_5_N_3_) ^+^∙

Found m/z= 631.3975

Spectrum of Compound **4f**, ^1^H NMR, 400MHz, CDCl_3_

__

Spectrum of Compound **4f** ^13^C NMR, JMOD, 100MHz, CDCl_3_

High resolution mass spectrum of compound **4f**, Tsource=70°C, Tprobe=320°C

Calculated m/z= 613.3874 (C38H51O4N3) +∙

Found m/z= 613.3871

Spectrum of Compound **5f**, ^1^H NMR, 400MHz, CDCl_3_

__

Spectrum of Compound **5f** ^13^C NMR, JMOD, 100MHz, CDCl_3_

High resolution mass spectrum of compound **5f**, Tsource=90°C, Tprobe=250°C

Calculated m/z=571.3768 (C_36_H_49_O_3_N_3_) ^+^∙

Found m/z=571.3774

Spectrum of Compound **3g**, ^1^H NMR, 500MHz, CDCl_3_

__

Spectrum of Compound **3g** ^13^C NMR, JMOD, 125MHz, CDCl_3_

High resolution mass spectrum of compound **3g**, Tsource=90°C, Tprobe=330°C

Calculated m/z=631.3980 (C_38_H_53_O_5_N_3_) ^+^∙

Found m/z=613.3868

Calculated m/z=613.3868 (C_38_H_51_O_4_N_3_) ^+^∙ [M–H_2_O]^+^

Spectrum of Compound **4g**, ^1^H NMR, 500MHz, CDCl_3_

__

Spectrum of Compound **4g** ^13^C NMR, JMOD, 125MHz, CDCl_3_

High resolution mass spectrum of compound **4g**, Tsource=100°C, Tprobe=320°C

Calculated m/z= 613.3874 (C_38_H_51_O_4_N_3_) ^+^∙

Found m/z= 613.3866

Spectrum of Compound **5g**, ^1^H NMR, 400MHz, CDCl_3_

__

Spectrum of Compound **5g** ^13^C NMR, JMOD, 100MHz, CDCl_3_

High resolution mass spectrum of compound **5g**, Tsource=90°C, Tprobe=330°C

Calculated m/z=571.3768 (C_36_H_49_O_3_N_3_) ^+^∙

Found m/z=571.3772

Spectrum of Compound **3h**, ^1^H NMR, 500MHz, CDCl_3_

__

Spectrum of Compound **3h** ^13^C NMR, JMOD, 125MHz, CDCl_3_

High resolution mass spectrum of compound **3h**, Tsource=90°C, Tprobe=280°C

Calculated m/z=631.3980 (C_38_H_53_O_5_N_3_) ^+^∙

Found m/z=613.3870 [M-H_2_O]^+^

Spectrum of Compound **4h**, ^1^H NMR, 500MHz, CDCl_3_

__

Spectrum of Compound **4h** ^13^C NMR, JMOD, 125MHz, CDCl_3_

High resolution mass spectrum of compound **4h**, Tsource=85°C, Tprobe=280°C

Calculated m/z= 613.3874 (C_38_H_51_O_4_N_3_) ^+^∙

Found m/z= 613.3871

Spectrum of Compound **5h**, ^1^H NMR, 400MHz, CDCl_3_

__

Spectrum of Compound **5h** ^13^C NMR, JMOD, 100MHz, CDCl_3_

High resolution mass spectrum of compound **5h**, Tsource=65°C, Tprobe=320°C

Calculated m/z= 571.3768 (C_36_H_49_O_3_N_3_)^+^

Found m/z= 571.3762
