# Supplementary material for: Novel 3′-Substituted-1′,2′,4′-Oxadiazole Derivatives of 18βH-Glycyrrhetinic Acid and Their O-Acylated Amidoximes: Synthesis and Evaluation of Antitumor and Anti-Inflammatory Potential In Vitro and In Vivo
Source: Int J Mol Sci. 2020 May 15;21(10):3511. doi: 10.3390/ijms21103511 (PMC7279002; doi:10.3390/ijms21103511)
Supplement: Supplementary file 1 [file ijms-21-03511-s001.zip › Supplementary Table 1.docx]

Novel 3ʹ-subsituted-1ʹ,2ʹ,4ʹ-oxadiazole derivatives of 18βH-glycyrrhetinic acid and their O-acylated amidooximes: synthesis and evaluation of anti-tumor and anti-inflammatory potential in vitro and in vivo

Andrey V. Markov ^1,^*, Aleksandra V. Sen’kova ^1^, Irina I. Popadyuk ^2^, Oksana V. Salomatina ^1,2^, Evgeniya B. Logashenko ^1^, Nina I. Komarova ^2^, Anna A. Ilyina ^1^, Nariman F. Salakhutdinov ^2^ and Marina A. Zenkova ^1^

^1^ Institute of Chemical Biology and Fundamental Medicine, Siberian Branch of the Russian Academy of Sciences, Lavrent’ev ave., 8, 630090 Novosibirsk, Russia; [alsenko@mail.ru](mailto:alsenko@mail.ru) (A.V.S.), [evg_log@niboch.nsc.ru](mailto:evg_log@niboch.nsc.ru) (E.B.L.), [humanity2206@mail.ru](mailto:humanity2206@mail.ru) (A.A.I.), [marzen@niboch.nsc.ru](mailto:marzen@niboch.nsc.ru) (M.A.Z.)

^2^ N.N. Vorozhtsov Novosibirsk Institute of Organic Chemistry, Siberian Branch of the Russian Academy of Sciences, Lavrent’ev ave., 9, 630090 Novosibirsk, Russia; [popadyuk@nioch.nsc.ru](mailto:popadyuk@nioch.nsc.ru) (I.I.P), [ana@nioch.nsc.ru](mailto:ana@nioch.nsc.ru) (O.V.S.), [komar@nioch.nsc.ru](mailto:komar@nioch.nsc.ru) (N.I.K.), [anvar@nioch.nsc.ru](mailto:anvar@nioch.nsc.ru) (N.F.S.)

***** Correspondence: [andmrkv@gmail.com](mailto:andmrkv@gmail.com) (A.V.M.); Tel.: +7-383-363-51-61

**Supplementary Table 1.** Biological functions of identified probable primary targets of **3d**

| ID | Name | Function^1^ |
| --- | --- | --- |
| Ache | Acetylcholinesterase | Neurotransmitter |
| Alox5 | Arachidonate 5-Lipoxygenase | Synthesis of leukotrienes from arachidonic acid, metabolism of fatty acid hydroperoxidases |
| Ca1 | Carbonic Anhydrase 1 | Catalysis of the reversible hydration of carbon dioxide; participates in a variety of biological processes, including respiration, calcification, acid-base balance, bone resorption, and the formation of aqueous humor, cerebrospinal fluid, saliva and gastric acid. |
| Ca2 | Carbonic Anhydrase 2 | Catalysis of the reversible hydration of carbon dioxide; essential for bone resorption and osteoclast differentiation |
| Ca9 | Carbonic Anhydrase 9 | Catalysis of the reversible hydration of carbon dioxide; participate in a variety of biological processes, including respiration, calcification, acid-base balance, bone resorption, and the formation of aqueous humor, cerebrospinal fluid, saliva, and gastric acid |
| Cnr2 | Cannabinoid Receptor 2 | Cannabinoid-induced CNS effects; may function in inflammatory response, nociceptive transmission and bone homeostasis. |
| Cyp17a1 | Cytochrome P450 Family 17 Subfamily A Member 1 | Catalysis of many reactions involved in drug metabolism and synthesis of cholesterol, steroids and other lipids. |
| Cyp19a1 | Cytochrome P450 Family 19 Subfamily A Member 1 | Catalysis of many reactions involved in drug metabolism and synthesis of cholesterol, steroids and other lipids. |
| Cyp3a4 | Cytochrome P450 Family 3 Subfamily A Member 4 | Catalysis of many reactions involved in drug metabolism and synthesis of cholesterol, steroids and other lipids. |
| Elane | Neutrophil Elastase | Serine protease; may play a role in degenerative and inflammatory diseases |
| F10 | Coagulation Factor X | Involved in blood coagulation cascade; converts prothrombin to thrombin in the presence of factor Va, calcium and phospholipid during blood clotting. |
| F2 | Thrombin | Involved in blood coagulation cascade; blood homeostasis, inflammation and wound healing |
| Hsd11b1 | Hydroxysteroid 11-Beta Dehydrogenase 1 | Catalysis of the conversion of the stress hormone cortisol to the inactive metabolite cortisone. |
| Hsd11b2 | Hydroxysteroid 11-Beta Dehydrogenase 2 | Catalyzes the conversion of cortisol to the inactive metabolite cortisone; modulates intracellular glucocorticoid levels, thus protecting the nonselective mineralocorticoid receptor from occupation by glucocorticoids |
| Ikbkb | Inhibitor Of Nuclear Factor Kappa B Kinase Subunit Beta | Immune response, growth control, or regulation of apoptosis |
| Mmp9 | Matrix Metalloproteinase 9 | Regulation of normal physiological processes, such as embryonic development, reproduction, and tissue remodeling, as well as in disease processes, such as arthritis and metastasis |
| Nr1h4 | Farnesoid X Receptor | Bile acid synthesis and transport |
| Ptges | Prostaglandin E Synthase | Catalyzes the oxidoreduction of prostaglandin endoperoxide H2 (PGH2) to prostaglandin E2 (PGE2) |
| Slco1b1 | Solute Carrier Organic Anion Transporter Family Member 1B1 | Mediates the sodium-independent uptake of numerous endogenous compounds including bilirubin, 17-beta-glucuronosyl estradiol and leukotriene C4 |
| Slco1b3 | Solute Carrier Organic Anion Transporter Family Member 1B3 | Mediates the sodium-independent uptake of endogenous and xenobiotic compounds and plays a critical role in bile acid and bilirubin transport. |

^1^ Functions of probable targets of **3d** were uploaded from GeneCards database (Weizmann Institute of Science, Israel)
